# Crohn's Disease—A Misnomer?

**Published:** 1988-02

**Authors:** Michael Harmer

**Affiliations:** Hon. Consulting Surgeon Royal Marsden Hospital

## Abstract

It is proposed that Regional Enteritis, commonly called Crohn's Disease should properly be named Crohn—Dalziel disease.

In October 1932 a 'Landmark Article' was published in the Journal of the American Medical Association entitled "Regional IIeitis, a pathologic and clinical entity." (1) It appeared below the names of Burrill B. Crohn, Leon Ginzburg and Gordon D. Oppenheimer. The first paragraphs of this article set the stage.


### Crohn's Disease – a Misnomer?

**Michael Harmer M.B., F.R.C.S.**
Hon. Consulting Surgeon Royal Marsden Hospital.

**SUMMARY**

It is proposed that Regional Enteritis, commonly called Crohn's Disease should properly be named Crohn–Dalziel disease.

In October 1932 a 'Landmark Article' was published in the Journal of the American Medical Association entitled "Regional Ileitis, a pathologic and clinical entity." (1) It appeared below the names of Burrill B. Crohn, Leon Ginzburg and Gordon D. Oppenheimer. The first paragraphs of this article set the stage.

> We propose to describe, in its pathological and clinical details, a disease of the terminal ileum, affecting mainly young adults, characterised by a subacute or chronic necrotising and cicatrising inflammation. The ulceration of the mucosa is accompanied by a disproportionate connective tissue reaction of the remaining walls of the involved intestine, a process which frequently leads to stenosis of the lumen of the intestine, associated with multiple fistulas. The disease is clinically featured by symptoms that resemble those of ulcerative colitis, namely fever, diarrhoea and emaciation, leading eventually to an obstruction of the small intestine. The constant occurrence of a mass in the right iliac fossa usually requires surgical intervention (resection). The terminal ileum is alone involved. The process begins abruptly at and involves the ileo–caecal valve in its maximum intensity, tapering off gradually as it ascends the ileum orally.... The etiology of the process is unknown, it belongs to none of the categories of recognised granulomatous or accepted inflammatory groups. The course is relatively benign, all the patients who survive operation being alive and well.... Such, in essence is the definition of a disease, the description of which is based on the study to date, of fourteen cases. These cases have been carefully observed and studied in their clinical course; the pathologic details have resulted from a close inspection of resected specimens from thirteen of fourteen patients operated on by Dr A. A. Berg.

Information about the events proceeding the 'Landmark paper' has been reported relatively recently in the J.A.M.A. by Kirsner (2) in 1984 and amplified by Ginzburg (3) in Gastro-enterology in 1986. Dr J. B. Kirsner related how in 1930 Dr Crohn examined two patients operated upon by his surgical colleague Dr A. A. Berg, and suggested to him that these might be reported. He did not take up the suggestion but he did tell Dr Crohn that two of his colleagues, Dr Leon Ginzburg and Dr Gordon Oppenheimer had already studied and described a series of 12 similar cases and he asked Dr Ginzburg to let Dr Crohn 'peruse' his already written article. Dr Ginzburg heard nothing more from Dr Crohn about the matter until it was discovered that Dr Crohn was intending to read a paper to a forthcoming meeting of the A.M.A. about the whole series under his own name only; and it was only on Dr Berg's insistence that Ginzburg and Oppenheimer's names were included.

Dr Berg himself declined to be included in the authorship on the laudable principle that he did not wish to put his name to anything he himself had not written. Had he agreed, under the conventional alphabetical authorship of multiple articles, the disease might have become known as Berg's disease.

Dr Ginzburg wrote his letter to Gastro-enterology "as the last surviving co-author of the original article on regional enteritis" with reluctance "in order to put the record straight" and to "deny emphatically certain assertions that Dr Crohn in his recently published memoir makes about the original investigations and descriptions of regional enteritis.". For the fact seems to be that Crohn may have taken credit for work done by others; certainly he basked in the aura of the eponym, though, in fairness it should be said that he preferred to call the condition ileitis or, as he was wont to pronounce it "eelieetis"; and that is not an inappropriate word to use because it recalls the description by T. K. Dalziel of the appearance of the terminal ileum as "an eel in rigor mortis"!

It is the purpose of this article to suggest that it is the Glasgow surgeon, Sir T. Kennedy Dalziel, who deserves the eponymous accolade. James Kyle (4) has put the case convincingly in quoting Dalziel's own words written 26 years before Crohn et al. (5). "I have pleasure in drawing your attention to a condition which has not yet I think been fully described". In his 89th year Dr Leon Ginzburg in reply to the writer's enquiry (6) took an even more robust view. "As to whether you dare call Regional Ileitis Dalziel's disease – my reply is – you should not only but *must.* Dalziel described the whole gamut of granulomatous disease of the bowel. Not only did he report Regional Enteritis but granulomatous colitis and the combined form involving both the large and the small bowel simultaneously".

Unquestionably Dalziel antedated Crohn as well as Moschowitz and Wilensky (7); yet even he may not have been aware that more than 100 years earlier, Dr William Saunders F.R.C.P. had communicated on behalf of Dr Charles Coombe "A singular case of stricture and thickening of the Ileum" in the Medical Transactions of the Royal College of Physicians (8). Though presented at the College in 1806, it did not appear in print for another seven years: but the plight of "William Payne Georges Esq. of very nervous and delicate habit [who] had been for many years troubled with flatulency and complaints in the bowels, attended with costiveness and a quick pulse" must strike a chord with us 180 years later, when we read that at post-mortem, "the lower part of the ileum as far as the colon was contracted for the space of three feet to the size of a turkey's quill," Mr Georges's death took place on Sunday morning, February 9th, 1806, "at which time he was more emaciated than any person we have ever witnessed". There seems little doubt that he suffered from regional ileitis, but from whose disease? Crohn's, Berg's Ginzburg's Wilensky's, Moschowitz's, Dalziel's, even Saunders's perhaps. And there are others too: Jackman recognised the disease in 1930 but failed to publish his findings until 1934 (9) thus missing the eponym by a whisker. Moynihan in 1907 (10) and Mayo Robson in 1908 (11) had discussed inflammatory tumours of the large bowel and so may also be considered. But as every searcher after truth knows, it is nearly always possible to discover an earlier reference and it is the conviction of the writer that one day a papyrus will come to light indicating that Imhotep, the true father of

Medicine, was well aware of the condition in 2,500 BC. Until that day arrives it is necessary to be practical.

It is both difficult and rash to suggest a change of name for an established syndrome; nevertheless there are a number of surgeons and pathologists both in Britain and the U.S.A. who believe that Dalziel has been given less credit that was his due. Armitage and Wilson (12) noted Dalziel's prior contribution and considered the question of nomenclature but nevertheless decided to adhere to the name Crohn's Disease because "it avoids confusion, makes no pretence of pathological exactitude, conveys an exact meaning, is easily remembered by students and pays a well deserved tribute".

I therefore suggest a compromise, in keeping with the many binomial or even trinomial diseases which adorn our literature and commemorate the pioneers of medicine – *Crohn–Dalziel disease*. Though Dalziel was first, placing his name second will obviate the confusion which might arise if his name is given precedence or if Crohn's name is eliminated altogether. Also Crohn–'Deeyell', to pronounce the name according to the Scottish usage, seems more euphonious than the other way round and therefore more acceptable.

Postscript: Dr Leon Ginzburg died on March 20th 1988.

### REFERENCES

1. CROHN, B. B., GINZBURG, L. and OPPENHEIMER, G. D. (1932) *J.A.M.A.* **99**, 1323–1329.
2. KIRSNER, J. B., (1984) Crohn's Disease. *J.A.M.A.* **251**, 80–81.
3. GINZBURG, L., (1986) Regional Enteritis, Historical perspective, *Gastroenterology.* **90**, 1310–1311.
4. KYLIE, J., (1979) Dalziel Disease—66 years on. *Br.Med.J.* (1979) **I**, 876–877.
5. DALZIEL, T. K., (1913) Chronic interstitial enteritis. *Br.Med. J.* **ii**, 1068–1070.
6. GINZBURG, L., (1986) Personal communication.
7. MOSCHOWITZ, E. and WILENSKY, A.O. (1923) Non-specific granuloma of the intestine. *Am.J.Med.Sci.* **166**, 48–66.
8. COOMBE, C. and SAUNDERS, W. (1813) Medical Transactions of the Royal College of Physicians. **4**, 16–21.
9. JACKMAN, W. A., (1934) *Br.J.Surg.* **22**, 27.
10. MOYNIHAN, B. G. A., (1907) *Edin.Med.J.* **21**, 229.
11. ROBSON, A. W. and MAY, O. (1980) *Br.Med.J.* **1**, 425.
12. ARMITAGE, G. A. and WILSON, M. G. (1950) Crohn's Disease. *Br.J.Surg.*, **38**, 182–193. [This paper contains the most beautiful hand paintings by Miss E. M. Wright of resected specimens].

